# The Effectiveness of Wearable Upper Limb Assistive Devices in Degenerative Neuromuscular Diseases: A Systematic Review and Meta-Analysis

**DOI:** 10.3389/fbioe.2019.00450

**Published:** 2020-01-24

**Authors:** Marta Gandolla, Alberto Antonietti, Valeria Longatelli, Alessandra Pedrocchi

**Affiliations:** ^1^Nearlab@Lecco, Polo Territoriale di Lecco, Politecnico di Milano, Lecco, Italy; ^2^Nearlab, Department of Electronics, Information and Bioengineering, Politecnico di Milano, Milan, Italy

**Keywords:** assistive devices, upper limbs, meta-analysis, wearable, neuromuscular disease

## Abstract

**Background:** This systematic review summarizes the current evidence about the effectiveness of wearable assistive technologies for upper limbs support during activities of daily living for individuals with neuromuscular diseases.

**Methods:** Fourteen studies have been included in the meta-analysis, involving 184 participants. All included studies compared patients ability to perform functional tasks with and without assistive devices.

**Results:** An overall effect size of 1.06 (95% CI = 0.76-1.36, *p* < 0.00001) was obtained, demonstrating that upper limbs assistive devices significantly improve the performance in activities of daily living in people with neuromuscular diseases. A significant interaction between studies evaluating functional improvement with externally-assessed outcome measures or self-perceived outcome measures has been detected. In particular, the effect size of the sub-group considering self-perceived scales was 1.38 (95% CI = 1.08-1.68), while the effect size of the other group was 0.77 (95% CI = 0.41-1.11), meaning that patients' perceived functional gain is often higher than the functional gain detectable through clinical scales.

**Conclusion:** Overall, the quality of the evidence ranged from low to moderate, due to low number of studies and participants, limitations in the selection of participants and in the blindness of outcome assessors, and risk of publication bias.

**Significance:** A large magnitude effect and a clear dose-response gradient were found, therefore, a strong recommendation, in favor of the use of assistive devices could be suggested.

## 1. Introduction

### 1.1. Background

Severe muscular weakness and chronic disability caused by neuromuscular diseases (e.g., muscular dystrophy, spinal muscular atrophy, spinal cord injuries or stroke) or neurodegenerative diseases (i.e., multiple sclerosis, amyotrophic lateral sclerosis) lead to the unavoidable loss of the possibility to perform even simple actions, such as walking, eating, and changing limbs posture. Patients suffer the consequences in terms of independence, quality of life, and self-esteem, given their need to continuously rely on assistance from their caregivers. This is particularly true for upper limbs, where independence is not primarily linked to essential tasks (e.g., eating, drinking, get dressed), but to simple actions not necessary for survival, but which increase the quality of life (e.g., pull up the glasses, scratch, use the mouse, etc.). To independently regain a lost motor function might be therefore a special experience towards a more independent daily life. Technological advancements might be a way to compensate patients' muscular weakness through the use of Assistive Devices (ADs), which empower the user in the execution of daily life activities, and which are designed to maintain or to improve the functional capabilities of individuals with disabilities. ADs for lower limbs, such as wheelchairs and electric wheelchairs, have been successfully developed and diffused to deal with the deambulation issue. On the other side, the support of upper limbs related activities is more challenging. However, with the increased life expectancy, upper extremities functions became more and more important to be supported. Non-ambulant patients with neuromuscular disorders identified arm functions as their highest priority, indicating repositioning at night, bring hands to mouth, shift while seated, using the wheelchair joystick and the keyboard of a computer, and personal hygiene as priority functions to be regained (Janssen et al., 2014). The currently existing assistive devices to support upper limbs functions can be categorized in (i) end-effector devices, and (ii) exoskeletons. As for end-effector devices, they present a single interaction point between the user and the AD, usually located at forearm or hand level. The main disadvantage of robotic manipulator devices is the impossibility to control upper limb joints directly: the change in position of the interaction point results in unexpected movements of shoulder and elbow joints. As for exoskeletons, they are external structures worn by the patient, with joints and links placed in correspondence of human joints and bones. Patients usually prefer exoskeleton solutions, given that these devices not only help to execute the desired task, but they increase the perception of a self-executed movement. In a study conducted by Rupal et al. (2017) with 118 participants, 96.8% prefer to use an exoskeleton over other mobility aids, and 84.1% like the idea that exoskeletons should be made available in care homes (Rupal et al., 2017). In addition, from a survey conducted by the authors at Lignano Sabbiadoro (Italy) on June 2015, during the annual meeting of the UILDM Association (Italian Association of Muscular Dystrophy), 10 out of 15 interviewed patients affected by muscular dystrophy answered that they prefer exoskeleton solutions for possible upper limbs assistive devices. ADs driving technology can be either passive, working through pre-stored mechanical energy, or active, working with motors, and therefore able to exert greater forces or to control movements more precisely. However, even if a remarkable number of works have been published dealing with the development of innovative electromechanical technologies (e.g., Ragonesi et al., 2013; Jung et al., 2014; Dunning et al., 2015; Sin et al., 2015; Dalla Gasperina et al., 2018), scientific evidence for the benefits of these technologies is still lacking, which could justify costs and effort. When dealing with Assistive Devices, or in general with complex technologies, the demonstration of the effectiveness of their use is rather difficult to be demonstrated following the canonical research studies design [i.e., Randomized Control Trial (RCT) design], even if some effort in this direction is currently ongoing (Antonietti et al., 2019). This is due to several reasons, such as the difficulty to demonstrate the validity of the proposed approach independently from the users' placebo effect (e.g., it is impossible to perform a blind session), the high cost of the technology and therefore the impossibility to recruit many volunteers contemporary, and the Ethical Committee procedures for non CE-marked devices. A recent systematic review on devices to assist and/or rehabilitate upper limbs made a quite large classification of different devices used, showing an intense research work towards the development of new technologies, which however are rarely methodologically properly tested, and therefore they have difficulties to effectively reach end-users (Onose et al., 2018).

### 1.2. Objectives

In this work, we propose a systematic evaluation of the available literature to assess the effectiveness and acceptability of ADs for individuals with neuromuscular diseases upper limbs functional improvement. In particular, the present study proposes a meta-analysis approach, that is the statistical analysis of a collection of analytic results for the purpose of integrating the findings. Indeed, combining the findings across studies represents an attractive alternative to strengthen the evidence about the treatment efficacy (DerSimonian and Laird, 1986). The overarching objective is to assess the short-term benefits of wearable devices for the activities of daily living and functional tasks, for people suffering from upper limb impairment due to degenerative neurological and neuro-motor diseases.

## 2. Methods

### 2.1. Criteria for Considering Studies for This Review

Criteria used for the inclusion of the studies in this analysis follow the PICOS format (Huang et al., 2006).

#### 2.1.1. Participants [P]

We included studies with participants affected by upper limb disability induced by neurological or neuromotor pathologies. The investigated pathologies include muscular dystrophy, spinal muscular atrophy, multiple sclerosis, amyotrophic lateral sclerosis, spinal cord injury, cerebral palsy or in general myopathies that leave patients with muscular weakness. No restrictions have been put in terms of age or sex.

#### 2.1.2. Types of Interventions [I]

We investigated the use of wearable assistive devices such as orthoses, prostheses, exoskeletons, electrical stimulation devices, and neuroprostheses with the aim to improve upper limbs activities of daily living. An example of an eligible exoskeleton is the Wilmington Robotic Exoskeleton (Wrex). It is a body-powered, four-degrees-of-freedom device that uses linear elastic bands for both balance and assistance of movement against the effects of gravity in three dimensions (Shank, 2017). The considered intervention is the execution of protocol-defined task(s) assisted by the AD.

#### 2.1.3. Types of Comparison Performed in the Included Studies [C]

The comparison has been performed with respect to the same task(s) executed during the intervention assessment, but without the use of the AD. In other words, each participant is him/herself part of both the intervention and comparison groups, given that s/he repeated the same functional task(s) both with (i.e., intervention) and without (i.e., control) the use of arm support (i.e., the AD).

#### 2.1.4. Types of Outcome Measures [O]

The primary outcome is the measurement of upper limb functional activity level as a comparison between use/non-use of the assistive device, either in terms of externally-assessed improvement (e.g., Fugl-Meyer Assessment scale, ARAT, Performance of the Upper Limb, and other specific scales, as applied by the therapist) or self-perceived functional improvement (e.g., ABILHAND, Canadian Occupational Performance Measure). The secondary outcomes were acceptance measures of the AD. To this aim, we used withdrawal or dropouts from the included studies due to any reason. We investigated the safety of different devices through the incidence of adverse outcomes, such as cardiovascular events, injuries and pain, and any other reported adverse events. Depending on the aforementioned categories and the availability of variables used in the included trials, all review authors discussed and reached consensus on which outcome measures should be included in the analysis.

#### 2.1.5. Types of Studies [S]

All designs of studies were accepted.

### 2.2. Search Methods for Identification of Studies

We restricted our searches to documents published between 2000 and 2018. No language restrictions were applied to reduce publication and retrieval bias.

#### 2.2.1. Electronic Searches

A comprehensive electronic search was performed to maximize the likelihood of identifying all eligible studies. In order to select all possible variants of keywords, a preliminary backward-forward references searching was performed. Backward references searching involves identifying and examining the references cited in a selected article of interest. Forward references searching, instead, is when a researcher identifies and looks at the articles that cite the identified article of interest after it had been published. A citation tracking and references list screening of all pertinent articles were broadly performed. Keywords were identified for three categories: (i) pathologies; (ii) devices; (iii) section of the body involved in the intervention (Table 1). Medical Subject Headings and free text terms for neuromuscular diseases and ADs for upper limbs were used to capture all research articles in this area. Methodological search filters by designs of study and outcomes were not applied to avoid potential retrieval bias. Multiple searches were performed to check the influence of each keyword on the search and some adjustments were implemented in response to database engines differences. The keywords (Table 1) were combined using Boolean Operators (AND, OR) as in the following search string: OR/1-13 AND OR/14-26 AND OR/27-32 (Lefebvre et al., 2008). We searched in the following bibliographic databases:

- The Cochrane Central Register of Controlled Trials (CENTRAL)^1^;
- Web of Science^2^;
- PubMed and MEDLINE^3^;
- International prospective register of systematic reviews PROSPERO^4^;
- The Physiotherapy Evidence Database^5^.

**Table 1:** Keywords used for searching in electronic database.

**Pathologies**	**Devices**	**Body sections**
1. Neuromuscular disease	14. Assistive device	27. Upper extremity
2. Neurodegenerative disease	15. Self help device	28. Upper limb
3. Neuromotor disease	16. Home device	29. Arm
4. Muscle weakness	17. Wearable device	30. Forearm
5. Arm impairment	18. Exoskeleton	31. Shoulder
6. Muscular dystrophy	19. Orthotic device	32. Elbow
7. Multiple sclerosis	20. Robotic arm	
8. Amyotrophic lateral sclerosis	21. Dynamic arm support	
9. Spinal muscular atrophy	22. Electrical stimulation device	
10. Paresis	23. Neuroprosthesis	
11. Spinal cord injury	24. Gravity balancing	
12. Myopathy	25. External manipulator	
13. Neuropathy	26. Man-machine system	

#### 2.2.2. Searching Other Resources

In an effort to identify further trials not available in the major databases, we screened references lists of all relevant articles.

### 2.3. Data Collection and Analysis

#### 2.3.1. Selection of Studies

The search results were independently screened by two reviewers (MG, VL). Inclusion criteria were as follows: studies which (1) included assistive devices for the upper limbs with aim of supporting activities of daily living, (2) involved participants with neuromuscular or neuromotor diseases, and (3) used valid outcome measure(s) which assessed the same task(s) with and without the use of the device. Exclusion criteria were as follows. Studies which: (1) involved therapy sessions with the aim to permanently restore a lost motor function without further assistance of the device, as opposed to assistive device with the aim to substitute a lost motor function; (2) do not involve the use of patient's own arm (i.e., the device used is not worn on the participant upper limb and do not directly moves patients upper limb to perform a functional movement), (3) make use of implanted devices or (4) use devices that assist only the patients hand. The two authors independently read the titles and the abstracts of identified publications and eliminated obviously irrelevant studies. We obtained the full text for the remaining studies and, according to the predetermined inclusion and exclusion criteria, they where independently ranked by the two authors as relevant, potentially relevant and irrelevant. Discrepancies between reviewers were resolved through discussion between all authors.

#### 2.3.2. Data Extraction and Management

Data extraction from the included studies was completed by one of two reviewers (VL), then the second reviewer (MG) checked the accuracy and completeness of extracted data. Any identified discrepancies were discussed by all authors to ensure the accuracy of the extracted data. The data extracted from the included studies were: (i) participants (i.e., number of participants, age, sex, type of disease, inclusion and exclusion criteria); (ii) type of device used; (iii) primary outcome measure(s). Studies' authors were contacted to request more information, clarification, or missing data when needed.

#### 2.3.3. Assessment of Risk of Bias in Included Studies

The evaluation of the risk of bias in clinical trials is required to lower the probability to formulate incorrect decisions about treatment effects (Gluud, 2006), since a systematic error or a deviation from the truth in results or inferences (i.e., biases) can lead to either underestimation or overestimation of the true intervention effect (Higgins and Altman, 2008). In this work, the ROBINS-I tool (Risk Of Bias In Non-randomized Studies–of Interventions) has been used, as described in Sterne et al. (2016), particularly effective in evaluating risks of bias of studies that did not use randomization to allocate interventions. ROBINS-I fundamental underlying principle lays on the comparison between the risks of bias associated with the current evaluated study and a target RCT, hypothetically conducted on the same participants group, even if this RCT may not be feasible or ethical (Schünemann et al., 2018). The ROBINS-I tool includes the evaluation of seven domains through which bias might be introduced into a non-randomized study: (1) bias due to confounding; (2) bias in selection of participants into the study; (3) bias in classification of interventions; (4) bias due to deviations from intended interventions; (5) bias due to missing data; (6) bias in measurement of outcomes (or detection bias); (7) bias in selections of the reported results. The ROBINS-I tool includes a series of signaling questions within each domain in order to facilitate judgments about the risk. The categories for risk of bias judgments are Low risk, Moderate risk, Serious risk and Critical risk. The risk of bias is firstly judged for each domain, and then assessed overall across the study. Two authors independently assessed risk of bias of the included studies (VL, AA), with disagreements between reviewers resolved through discussion between all authors. Two authors (MG and AP) were co-authors of one included trial (Ambrosini et al., 2014); and they did not participate to the quality assessment for this study.

#### 2.3.4. Measures of Treatment Effect

The primary outcome variables of interest were treated as continuous data and entered as means (*m*_*i*_) and standard deviations (σ_*i*_), where *i* is the index for each included study. Since the studies assessed the same outcome (i.e., upper limbs functional improvement), but measured it with different outcome measures, the standardized mean difference (*SMD*_*i*_) with 95% confidence interval (*CI*_*i*_) was used as summary statistic, in order to standardize the results of the studies to a uniform scale before they can be combined. The *SMD*_*i*_ expresses the size of the intervention effect in each study, relatively to the observed variability (Deeks et al., 2008), and it is expressed as the difference in mean outcome between groups divided by the pooled standard deviation of outcome among participants (Lakens, 2013), as follows (Equation 1):

(1) $$\begin{array}{l} SMD_{i}=\frac{m_{Ti}-m_{Ci}}{\sqrt{\frac{\left(N_{Ti}-1\right)\sigma_{Ti}^{2}\;+\;\left(N_{Ci}-1\right)\sigma_{Ci}^{2}}{N_{Ti}\;+\;N_{Ci}-2}}} \end{array}$$

where *m*_*T*_, σ_*T*_, *N*_*T*_ and *m*_*C*_, σ_*C*_, *N*_*C*_ are the mean outcome, the standard deviation and the sample size of the Treatment (T) and the Control (C) group, respectively. A *SMD*_*i*_ of zero means that the treatment and the control group have equivalent effects. Since improvements are associated with higher scores on the outcome measures, *SMD*_*i*_ > 0 indicates the degree to which treatment is more effective than control, and *SMD*_*i*_ < 0 indicates the degree to which treatment is less effective than control.

This *SMD*_*i*_ (Equation 1) overestimates population effect sizes when sample sizes are small, and therefore it was corrected with the Hedges' g as follows (Equation 2) (Hedges and Olkin, 1985):

(2) $$\begin{array}{l} g_{i}=SMD_{i}\cdot \left(1-\frac{3}{4\cdot \left(N_{Ti}+N_{Ci}\right)-9}\right) \end{array}$$

The within-group standard deviation of *g* has been calculated as standard error (*SE*_*i*_) as follows (Equation 3) (Hedges and Olkin, 1985):

(3) $$\begin{array}{l} SE_{i}=\sqrt{\frac{N_{Ti}+N_{Ci}}{N_{Ti}\cdot N_{Ci}}+\frac{g_{i}^{2}}{2\cdot \left(N_{Ti}+N_{Ci}\right)}} \end{array}$$

The confidence interval *CI*_*i*_ for each *g*_*i*_ was given by Equation (4) (Hedges and Olkin, 1985):

(4) $$\begin{array}{l} CI_{i}=g_{i}\pm z_{1-\frac{\alpha }{2}}\cdot SE_{i} \end{array}$$

where $z_{1-\frac{\alpha }{2}}$ is the $1-\frac{\alpha }{2}$ quartile of the normal distribution. In this meta-analysis, α was set to 0.05.

In the second stage of the meta-analysis, a summary intervention effect estimate θ was calculated as a weighted average of the intervention effects estimated in the individual studies defined as in Equation (5) (Marín-Martínez and Sánchez-Meca, 2010):

(5) $$\begin{array}{l} \theta =\frac{\sum_{i=1}^{k}g_{i}\cdot w_{i}}{\sum_{i=1}^{k}w_{i}} \end{array}$$

where *g*_*i*_ is the intervention effect estimated in the *i*^*th*^ study, *w*_*i*_ is the weight given to the *i*^*th*^ study, and *k* indicates the number of studies included in the analysis.

Weights were estimated with the inverse-variance method (Deeks et al., 2008). Thus, studies with larger sample sizes, which have smaller standard errors, are weighted more than studies with smaller sample sizes, which have larger standard errors. This choice minimizes the imprecision (uncertainty) of the estimated effect size, and it is obtained as follows (Equation 6):

(6) $$\begin{array}{l} w_{i}=\frac{1}{\tau^{2}+SE_{i}^{2}} \end{array}$$

where τ^2^ is the between-study variance derived with the method proposed by DerSimonian and Laird (Equation 7) (DerSimonian and Laird, 1986):

(7) $$\begin{array}{l} \tau^{2}=\frac{Q-\left(k-1\right)}{c}; \end{array}$$

*Q* is the heterogeneity statistic (Equation 12), and *c* is given by Equation (8):

(8) $$\begin{array}{l} c=\overset{k}{\underset{i=1}{\sum }}w_{i}^{FE}-\frac{\sum_{i=1}^{k}{w_{i}^{FE}}^{2}}{\sum_{i=1}^{k}w_{i}^{FE}}. \end{array}$$

$w_{i}^{FE}$ is the weighting factor for the *i*^*th*^ study assuming a fixed-effects model ($w_{i}^{FE}=\frac{1}{\sigma_{i}^{2}}$) (Marín-Martínez and Sánchez-Meca, 2010). The standard error (*SE*) of the summary intervention effect was then computed as (Equation 9):

(9) $$\begin{array}{l} SE\left(\theta \right)=\frac{1}{\sqrt{\sum_{i=1}^{k}w_{i}}} \end{array}$$

Finally, the two-sided confidence interval for θ was obtained with the following (Equation 10), with α = 0.05:

(10) $$\begin{array}{l} CI_{\theta }=\theta \pm z_{1-\frac{\alpha }{2}}\cdot SE_{\theta } \end{array}$$

If a study included more than an outcome measure that assessed upper limb functionality (e.g., range of motion at shoulder, elbow and wrist levels) or includes two or more groups, sample sizes, means and standard deviations were properly merged, as detailed in Table 2 (Deeks et al., 2008).

**Table 2:** Formulas for merging data of two groups.

	**G1**	**G2**	**G1 + G2**
Sample Size	*N*_1_	*N*_2_	*N*_1_ + *N*_2_
Mean Value	*m*_1_	*m*_2_	$\frac{N_{1}m_{1}\;+\;N_{2}m_{2}}{N_{1}\;+\;N_{2}}$
Standard Deviation	σ_1_	σ_2_	$\sqrt{\frac{\left(N_{1}-1\right)\sigma_{1}^{2}\;+\;\left(N_{2}-1\right)\sigma_{2}^{2}\;+\;\frac{N_{1}N_{2}}{N_{1}\;+\;N_{2}}\left(m_{1}^{2}\;+\;m_{2}^{2}\;-\;2m_{1}m_{2}\right)}{N_{1}\;+\;N_{2}-1}}$

#### 2.3.5. Measures of Acceptance

Participants' acceptability of the ADs was investigated analyzing dropouts and withdrawal through the risk difference index (*RD*) along with its corresponding 95% *CI*. The risk difference is the difference between the proportions of individuals with the effective assessment of the outcome of interest (i.e., observed risks) in the treatment group and in the control group (Equation 11) (Deeks et al., 2008):

(11) $$\begin{array}{l} RD=\frac{S_{T}}{N_{T}}-\frac{S_{C}}{N_{C}} \end{array}$$

where *S*_*T*_ and *S*_*C*_ are the number of dropouts and withdrawal in the treatment (T) and in the control group (C) respectively, while *N*_*T*_ and *N*_*C*_ represent the total number of participants in each group.

#### 2.3.6. Assessment of Heterogeneity

Between-studies variability (i.e., differences among the population effect sizes estimated by individual studies) might be a source of heterogeneity. If there is statistically-significance between-studies heterogeneity, moderator variables can be examined to explain this variability (e.g., participants characteristics, outcome measures, treatment conditions, study designs, etc.) (Huedo-Medina et al., 2006). The statistical test usually applied in meta-analysis for determining whether there is true heterogeneity among study effects sizes is the *Q* test, proposed by Cochran (Cochran, 1954; Hedges and Olkin, 1985) and defined as (Equation 12):

(12) $$\begin{array}{l} Q=\overset{k}{\underset{i=1}{\sum }}w_{i}^{FE}-{\left(g_{i}-ES^{FE}\right)}^{2} \end{array}$$

where *ES*^*FE*^ is the effect size assuming a fixed effect model (i.e., Equation 5, with τ = 0). A shortcoming of the *Q* statistic is that it has poor power to detect true heterogeneity among studies, when the meta-analysis includes a small number of studies (Huedo-Medina et al., 2006; Fletcher, 2007), as in the current case. To solve this issue, Higgins and Thompson (2002) have proposed the *I*^2^ index, which quantifies the extent of heterogeneity from a collection of effect sizes by comparing the *Q* value to its expected value assuming homogeneity, that is to its degrees of freedom (*df* = *k* − 1), as in Equation (13):

(13) $$\begin{array}{l} I^{2}=\frac{Q-\left(k-1\right)}{Q}\cdot 100 \end{array}$$

The *I*^2^ index can be interpreted as the percentage of the total variability in a set of effect sizes due to true heterogeneity (i.e., between-studies variability) rather than due to sampling error (i.e., chance). *I*^2^ ranges from 0 to 100%, with 0% indicating that statistical homogeneity exists. Higgins and Thompson (Higgins and Thompson, 2002) proposed a tentative classification of *I*^2^ values with the purpose of helping to interpret its magnitude. Percentages of around 25% (*I*^2^ = 25), 50% (*I*^2^ = 50), and 75% (*I*^2^ = 75) would mean low, medium, and high (Sedgwick, 2013). Therefore, if a cluster of studies presents a characteristic of non-homogeneity, it is worthy to investigate its source.

#### 2.3.7. Sub-groups Analysis and Investigation of Heterogeneity

Given an expected heterogeneity between the studies involved in this research, two sub-groups analyses were hypothesized. As a first hypothesis, the studies were split into two sub-groups according to the type of device involved: (i) studies that involved a passive device [i.e., non-actuated or passively actuated with counterweights, springs, or elastic bands to passively compensate for the impact of gravity on the arm (Van der Heide et al., 2014)] and (ii) studies performed with an active device (i.e., not dependent on pre-stored mechanical energy, equipped with sensors and actuators). If a study involved the use of both active and passive devices, it has been split into two sub-studies: one including the patients that used passive and one with patients that used active devices.

Another possible source of heterogeneity was identified by the type of primary outcome measure employed, and namely: (i) studies characterized by a self-perceived outcome measure (i.e., outcome measures derived by the perception of the patient him/herself according, for example, to the perceived functional benefit or the difficulty in accomplishing an exercise, e.g., Canadian Occupational Performance Measure) and (ii) studies with externally-assessed outcome measures (i.e., all rigorous and methodological scales performed by clinicians or through a system measurement, e.g., Fugl-Meyer scale, range of motion).

Within the sub-groups analyses, treatment effects in sub-groups have been compared by a test of interaction that investigated whether the effect size of the intervention in the primary outcome measure varied between the sub-groups. In particular, for both sub-groups analyses, differences in treatment effects have been evaluated with the *I*^2^ (Equation 13). In particular, *I*^2^ = 0% indicates no effect between sub-groups (Sedgwick, 2013).

#### 2.3.8. Data Synthesis

The results of all eligible studies were pooled in order to present an overall estimate of the effect of ADs in supporting activities of daily living. For all statistical analyses, Cochrane Review Manager software (RevMan 5.3) was used. We calculated the overall effect size using a random-effects model, regardless of the level of heterogeneity (Mehrholz et al., 2015). Clinical diversity and heterogeneity did not contribute to the decision on when to pool trials, but we described clinical diversity, variability in participants, interventions, and outcomes studied in Tables 3, 4. A *z*-test was applied to overall estimates of the effect sizes with the null hypothesis that there was no statistically significant difference between the treatment (i.e., users using AD) and the control group (i.e., users not using AD). The null hypothesis of no statistically difference was rejected if *p*-values were <0.05.

**Table 3:** Details of study interventions.

**Study ID**	**N**	**Outcome measure**	**Device**	**Mounting**
Gunn et al. (2016)	55	Five-point Likert scale	Passive (Wrex)	Attached to the wheelchair or to a body jacket
Shank (2017)	25	COPM	Passive (Wrex)	Attached to the wheelchair or worn by the patient
van der Heide and de Witte (2016)	19	Perceived functional benefit	Active (Armon Edero, Armon Ayura, Darwing) or Passive (Balancer)	Attached to the wheelchair, chair or table
Peters et al. (2017)	18	Fugl-Meyer scale	Active (EMyoPro Motion-G and powered orthosis)	Worn by the patient on wheelchair
Rahman et al. (2007)	13	Jebsen Hand Function Test	Passive (Wrex)	Attached to the wheelchair
Iwamuro et al. (2008)	10	FOR	Passive (T-Wrex)	Attached to the wheelchair
Estilow et al. (2018)	9	ROM	Passive (Wrex)	Attached to the wheelchair
Jan Burgers (2015)	8	ARAT	Active (Top/Help Electrical) or Passive (Top/Help Mechanical)	Attached to the wheelchair
Lund et al. (2009)	7	IPPA	Active (Armon)	Attached to the wheelchair or placed on a floor stand
Sanchez et al. (2004)	5	Fugl-Meyer scale	Passive (T-Wrex)	Attached to the wheelchair
van der Heide et al. (2017)	5	ROM	Active (Top/Help) or Passive (Sling)	Attached to the wheelchair
Bastiaens et al. (2011)	4	ROM	Active (HapticMaster and Sling)	Attached to the wheelchair
Ambrosini et al. (2014)	3	RMSE	Active (NMES and passive exoskeleton)	Attached to the wheelchair
Kooren et al. (2015)	3	PUL	Passive (A-gear)	Worn by the patient on wheelchair

*N, number of participants in each study; ARAT, Action Research Arm Test; COPM, Canadian Occupational Performance Measure; FOR, Fraction Of Reach; IPPA, Individually Prioritized Problem Assessment; PUL, Performance of the Upper Limb; RMSE, Root Mean Square Error; ROM, Range Of Motion*.

**Table 4:** Participants' characteristics in studies.

**Study ID**	**Diseases**	**Age Mean (SD)**	**Sex**	**Baseline characteristics Mean (SD)**
Gunn et al. (2016)	AMC (27), CP (5), MD (8), SMA (9), others (6)	9 (6)	—	—
Shank (2017)	AMC (14), CP (3), MD (2), SMA (2), others (4)	8 (1)	9 F, 16 M	—
van der Heide and de Witte (2016)	ALS (1), MD (1), MS (3), SCI (1), SMA (8), others (5)	55 (15)	11 F, 8 M	3.4 (1.7) points in Brooke scale
Peters et al. (2017)	Stroke (18)	56 (12)	7 F, 11 M	—
Rahman et al. (2007)	MD (10), SMA (3)	13 (4)	1 F, 12 M	3.3 (0.7) points in MMT
Iwamuro et al. (2008)	Chronic hemiparesis after stroke (10)	58 (14)	5 F, 5 M	—
Estilow et al. (2018)	DMD (9)	15 (2)	9 M	4.5 (0.7) points in Brooke scale
Jan Burgers (2015)	DMD (8)	15 (3)	8 M	3.8 (1.0) points in Brooke scale
Lund et al. (2009)	ALS (2), AMC (1), MD (2), SMA (2)	—	5 F, 2 M	—
Sanchez et al. (2004)	Chronic stroke (5)	—	—	—
van der Heide et al. (2017)	ALS (1), MD (1), SCI (1), Stroke (1), others (1)	52 (15)	3 F, 2 M	3.4 (0.9) points in Brooke scale
Bastiaens et al. (2011)	MS (4)	57 (8)	—	35.8 (20.8) points in MI
Ambrosini et al. (2014)	SCI (3)	51 (19)	3 M	43.7 (15.8) points in MI
Kooren et al. (2015)	DMD (3)	15 (2)	3 M	2.6 (0.6) points in Brooke scale

*AMC, Arthrogryposis Multiplex Congenital; ALS, Amyotrophic Lateral Sclerosis; CP, Cerebral Palsy; DMD, Duchenne Muscular Dystrophy; MD, Muscular Dystrophy; MS, Multiple Sclerosis; SCI, Spinal Cortex Injury; SMA, Spinal Muscular Atrophy; M, Male; F, Female; MMT, Manual Muscle Test; MI, Motricity Index*.

### 2.4. Quality of Evidence Assessment

The overall quality of the evidence and strength of recommendation was assessed by the Grades of Recommendation Assessment, Development and Evaluation (GRADE) methodology (Atkins et al., 2004). The GRADE approach specifies four levels of quality: (i) high, (ii) moderate, (iii) low and (iv) very low. In addition, it expresses the confidence that the estimated effect size lies close to the true effect, and the extent to which it is believed to be stable based on the adequacy or deficiencies in the body of evidence. The GRADE assignment starts from high (Sterne et al., 2016; Schünemann et al., 2018), and is downgraded, based on the following criteria: (a) risk of bias; (b) indirectness of evidence; (c) unexplained heterogeneity or inconsistency of results; (d) imprecision of results, and (e) high probability of publication bias. In addition, there are two factors that may lead to one level upgrade of the evidence (Guyatt et al., 2011): (a) large magnitude of effect; and (b) clear dose-response gradient. An overall strength of “high” means that considered studies report consistent and precise data, therefore that the true effect lies close to the estimated one. A “very low” rating means that a high level of uncertainty arises from current evidence and the true effect may substantially be different from the estimated findings. Based on the body of evidence, it could be possible to appraise the strength of recommendations for clinical practice. The GRADE approach classifies recommendations as strong, moderate, weak and not to do. Information related to the quality of evidence and the strength of recommendations could be summarized using a 4-level color schema, as illustrated in Table 5. This schema, suggested by Kremer and colleagues (Kremer et al., 2013), adapts the general guidelines offered by the American Heart Association/American Stroke Association for the classification of recommendations based on the level of evidence (Winstein et al., 2016).

**Table 5:** Four-levels color schema in order to integrate the quality of evidence assessment and the strength of recommendations.

**Level of evidence**	**High**	**Moderate**	**Low**	**Very low**
**Grade of recommendation**	*Consistent evidence from well performed and high quality studies or systematic reviews*	*Evidence from studies or systematic reviews with few important limitations*	*Evidence from studies or systematic reviews with some important limitations*	*Evidence from studies with serious flaws*
**Class I–Strong recommendation to do** *Benefits >>> risk*	Strong recommendation based on high level of evidence	Strong recommendation based on moderate level of evidence	Strong recommendation based on low level of evidence	Strong recommendation based on expert opinion
**Class IIa–Moderate recommendation to do** *Benefits >> risk*	Moderate recommendation based on high level of evidence	Moderate recommendation based on moderate level of evidence	Moderate recommendation based on low level of evidence	Moderate recommendation based on very low level of evidence; diverging expert opinions
**Class IIb–Weak recommendation to do** *Benefits ≥ risks*	Weak recommendation based on high level of evidence	Weak recommendation based on moderate level of evidence	Weak recommendation based on low level of evidence	Weak recommendation based on very low level of evidence; diverging expert opinion
**Class III–Recommendation not to do** *No benefit/Potentially harm*	Recommendation based on high level of evidence	Recommendation based on moderate level of evidence	Recommendation based on low level of evidence	Recommendation based on very low level of evidence

*Colors represent the strength of recommendation regarding the investigated intervention (i.e., use of AD) based on the level of evidence. Green, yellow and orange represent strong, moderate and weak recommendation to use AD, respectively, while red means recommendation not to use AD*.

## 3. Results

### 3.1. Results of the Search

**Figure 1:**
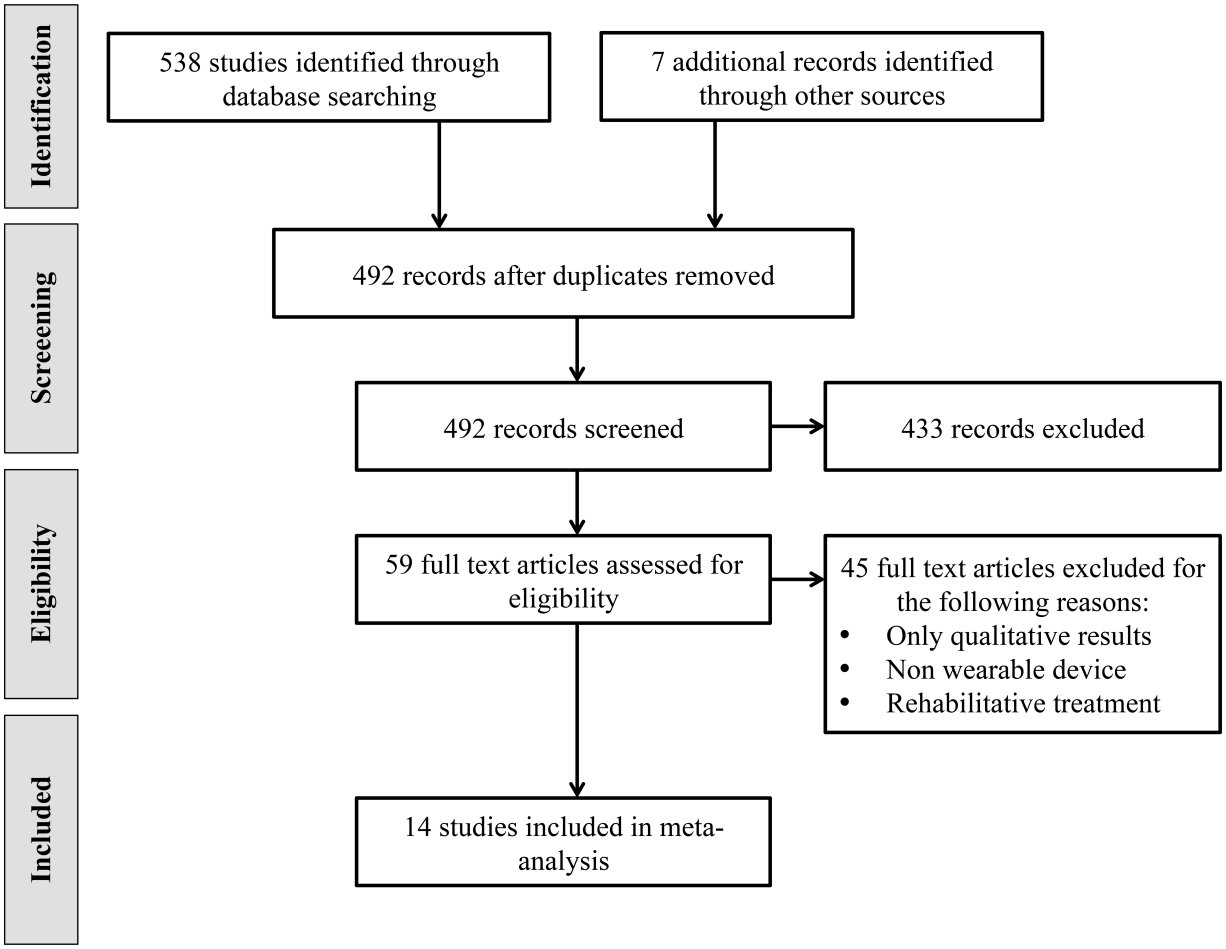
PRISMA flowchart of the literature search process.

The studies selection process was performed in accordance with the Preferred Reporting Items for Systematic Reviews and Meta-analysis (PRISMA) statements (Moher et al., 2009). A flow chart outlining the studies selection process is shown in Figure 1. The electronic databases search resulted in a total of 538 identified studies. Additional searches conducted on trials registers, commercial web-sites, conference proceedings and references lists resulted in 7 additional studies. The total number of records was therefore 545. After the removal of 53 duplicates, review authors assessed relevant abstracts and eliminated obviously irrelevant studies from the titles and abstracts alone. The full texts of 59 studies were obtained. The same authors independently reviewed the full papers and selected 20 studies that met inclusion criteria. 6 of these studies were excluded because they reported only qualitative results. Thus, 14 studies have been identified and included in this meta-analysis.

### 3.2. Characteristics of Included Studies

Fourteen trials, including a total of 184 participants, met inclusion criteria and have been included in the analysis. Each participant took part to both the treatment group (i.e., functional evaluation with the device) and the control group (i.e., functional evaluation without the device). Characteristics of included studies are summarized in Table 3.

#### 3.2.1. Sample Sizes

The sample sizes in the trials ranged from three participants, in Ambrosini et al. (2014);Kooren et al. (2015), to 55 participants, in Gunn et al. (2016). The median of the sample size is 9, with 13 as interquartile range. Sample sizes for all included studies are detailed in Table 3.

#### 3.2.2. Participants

The characteristics of the 184 participants, grouped for each study, are listed in Table 4. The mean age of participants in the included studies ranged from 8 years, in Shank (2017), to 58 years, in Iwamuro et al. (2008). The percentage of involved males was higher (65% with 95% *CI*= [63.8%-66%]). Participants across all trials had different diagnoses of neuromuscular diseases. The mostly investigated disease was Muscular Dystrophy (MD), included in 9/14 trials, and involving the 24% of total patients. Other included pathologies were Arthrogryposis Multiplex Congenital (AMC), Stroke resulting in hemiparesis (i.e., hemiparesis stroke), Spinal Muscular Atrophy (SMA), Hemiparesis, Cerebral Palsy (CP), Amyotrophic Lateral Sclerosis (ALS), Multiple Sclerosis (MS), and Spinal Cord Injury (SCI). Only eight studies (Rahman et al., 2007; Bastiaens et al., 2011; Ambrosini et al., 2014; Jan Burgers, 2015; Kooren et al., 2015; van der Heide and de Witte, 2016; van der Heide et al., 2017; Estilow et al., 2018) provided information about baseline deficit of arm motor function through the Brooke scale (Brooke et al., 1981), the Manual Muscle Test (Ciesla et al., 2011) or the Motricity Index (Bohannon, 1999). Mean values and standard deviations are reported in Table 4. For inclusion and exclusion criteria of each included study, see Characteristics of included studies in Supplementary Material.

#### 3.2.3. Interventions

All included studies involved the use of a wearable upper limb AD, as specified in the studies inclusion criteria. The included ADs are detailed for each included study in Table 3. Four studies concerned the use of an active AD (Lund et al., 2009; Bastiaens et al., 2011; Ambrosini et al., 2014; Peters et al., 2017), 7 investigated the use of passive ADs (Sanchez et al., 2004; Rahman et al., 2007; Iwamuro et al., 2008; Kooren et al., 2015; Gunn et al., 2016; Shank, 2017; Estilow et al., 2018), and 3 investigated the use of both active and passive ADs (Jan Burgers, 2015; van der Heide and de Witte, 2016; van der Heide et al., 2017). The most used (passive) AD was Wrex, which was employed in six studies (Sanchez et al., 2004; Rahman et al., 2007; Iwamuro et al., 2008; Gunn et al., 2016; Shank, 2017; Estilow et al., 2018). Participants were able to familiarize or to use the device for a variable period of time, ranging from a single session in a controlled environment such as the clinical setting (Sanchez et al., 2004; Iwamuro et al., 2008; Bastiaens et al., 2011; Ambrosini et al., 2014; Jan Burgers, 2015; Kooren et al., 2015; Peters et al., 2017; Estilow et al., 2018), to regular use at home for a mean range of four months to 25 months (Lund et al., 2009; Gunn et al., 2016; van der Heide and de Witte, 2016; Shank, 2017). However, the outcome measure assessment with and without the device did have a maximum inter-timing of 2 weeks (Shank, 2017).

#### 3.2.4. Comparisons

The included trials compared the ability of the patient to perform activities of daily living with and without AD assistance. The intensity of treatment (in terms of duration of use of the AD) ranged from one single session in one day (Sanchez et al., 2004; Iwamuro et al., 2008; Bastiaens et al., 2011; Ambrosini et al., 2014; Jan Burgers, 2015; Kooren et al., 2015; Peters et al., 2017; Estilow et al., 2018), up to a mean of 25 months of regularly use of the AD (Shank, 2017). A detailed description of comparison is provided in Characteristics of included studies in Supplementary Material.

#### 3.2.5. Outcome Measures

Primary outcome measures used in the included studies, and analyzed in this work are detailed in Table 3. Possible secondary outcome measure(s) which may have been assessed have not been included in this study. Analyzed outcome measures included self-perceived outcome measures, and externally-assessed outcome measures. We classified as self-perceived the following outcome measures. (i) Five-point Likert scale (Gunn et al., 2016), where patients are asked to answer to ten questions on their functional ability without and with the use of the AD. The answers ranged from “performing the task without any difficulty” to “unable to do it.” (ii) Canadian Occupational Performance Measure (Shank, 2017), where patients are asked to evaluate their ability to perform 5 selected activities of daily living from 1 (“completely unable to perform it”) to 10 (“able to perform it very well”). (iii) Perceived Functional Benefit (van der Heide and de Witte, 2016), computed as the difference in patients self-evaluation of ability to perform activities of daily living with and without the AD in 51 items, and (iv) the Individually Prioritized Problem Assessment (IPPA) (Lund et al., 2009). On the other hand, the included externally-assessed outcome measures were the following. (i) Upper extremity section of Fugl-Meyer scale (Sanchez et al., 2004; Peters et al., 2017), designed to assess motor functioning, balance, sensation and joint functioning in post-stroke patients. (ii) Jebsen Hand Function Test (Rahman et al., 2007), which calculates the completion time of seven specific upper limbs tasks (e.g., writing or moving light and heavy objects). (iii) Fraction Of Reach scale (Iwamuro et al., 2008), computed by comparing the minimum distance to the target achieved by the participant to the Euclidean distance between the starting hand posture and the target, while performing reaching of objects placed in 12 different positions at the limits of the patient-specific reaching space. (iv) Range Of Motion (ROM) of the shoulder and elbow joints (Bastiaens et al., 2011; van der Heide et al., 2017; Estilow et al., 2018), (v) Action Research Arm Test (ARAT) (Jan Burgers, 2015), (vi) the Root Mean Square Error (RMSE) between the actual and the target angle (Ambrosini et al., 2014). (vii) Performance of the Upper Limb scale (PUL) (Kooren et al., 2015), a new tool recently designed for specifically assessing arm functionality in DMD patients through 22 items at the shoulder, elbow and wrist/fingers levels.

#### 3.2.6. Design of Studies

All included studies were cohort studies according to the definition proposed by Mathes and colleagues (Mathes and Pieper, 2017), i.e., studies that contain sufficient data to conduct a re-analysis and thus are appropriate for inclusion in systematic reviews.

### 3.3. Assessment of Risk of Bias in Included Studies

**Figure 2:**
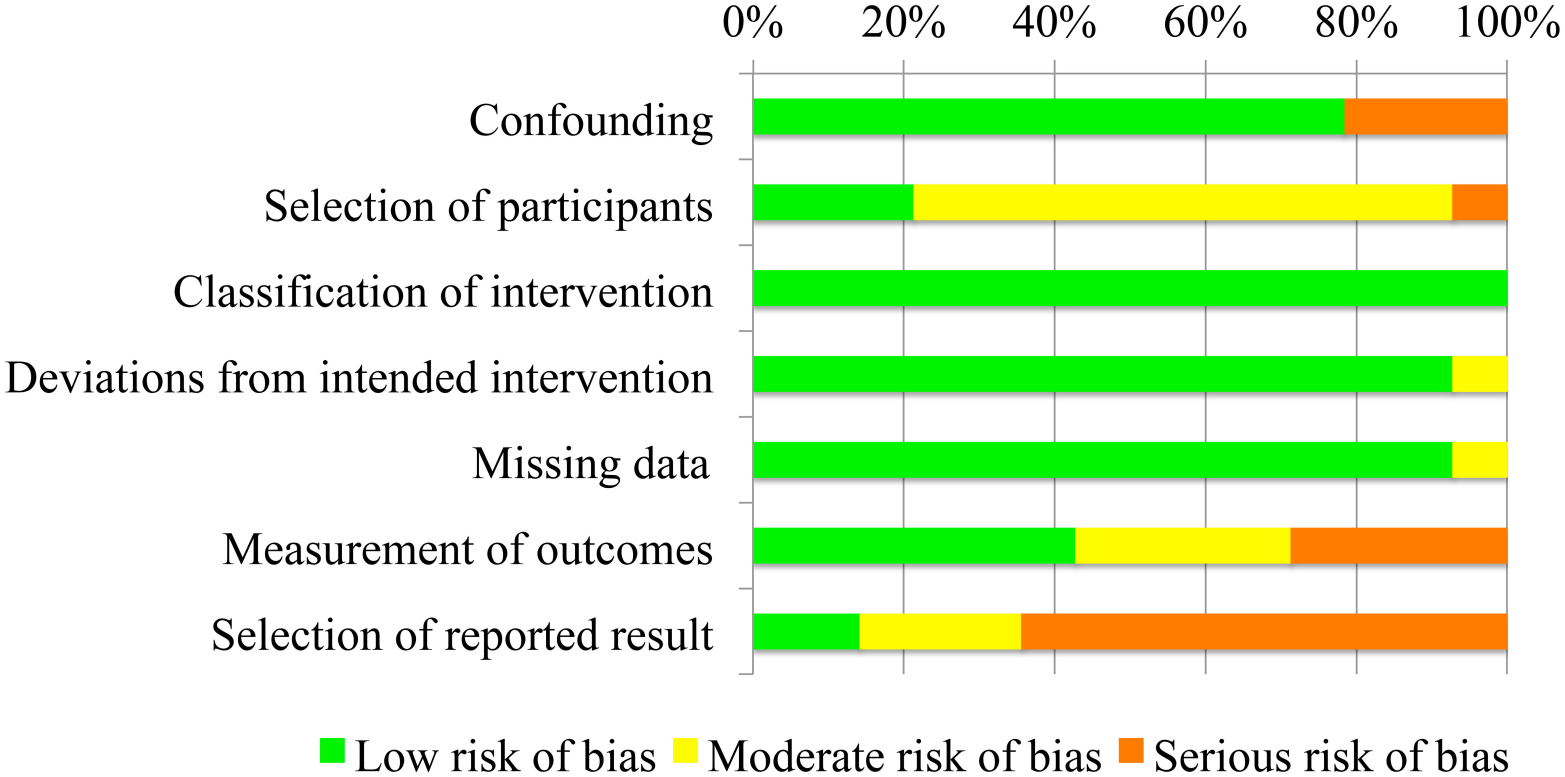
Risk of bias of the fourteen included studies presented as percentages across all included studies.

Risks of bias represented as percentage across all included studies are shown in Figure 2. Following the ROBINS-I tool, the risks of bias have been classified as follows:

- Confounding bias—all studies presented a low confounding associate risk, except for (Lund et al., 2009; Gunn et al., 2016; Shank, 2017), where enrolled patients already regularly used the AD at least in the last 4 months.
- Selection of participants bias—the risk in selection of participants was low for three studies (Iwamuro et al., 2008; Peters et al., 2017; Estilow et al., 2018), since they were characterized by precise inclusion criteria and all patients who would have been eligible for the trial were enrolled in the study. The selection of participants risk of bias was classified as moderate for ten studies (Sanchez et al., 2004; Rahman et al., 2007; Lund et al., 2009; Bastiaens et al., 2011; Ambrosini et al., 2014; Janssen et al., 2014; Kooren et al., 2015; Gunn et al., 2016; van der Heide and de Witte, 2016; Shank, 2017), where selection into the study may have been related to intervention and outcome, since no inclusion criteria were present. Van Der Heide and colleagues study in 2017 (van der Heide et al., 2017) presented a serious risk of bias, since this study explicitly says that “participants were selected on the basis of convenience sampling.”
- Classification of intervention bias—data were collected at the time of the intervention. In each study, all patients underwent the same protocol and test without and with the AD, and the outcome measures were immediately recorded. Classification of intervention risk of bias has been therefore classified as low for all studies.
- Deviations from intended interventions bias - one single study (Rahman et al., 2007) presented a deviation from intended intervention, since few patients did not practice at home with the AD, as they have been told by the therapists. However, the impact of this deviation from the intended treatment was considered to have a moderate impact on the outcome measure.
- Missing data bias—only one study (Rahman et al., 2007) was characterized by a moderate risk of bias, since proportions of missing participants differed slightly across intervention and control groups and, in particular, it was higher in the intervention group (see 3.5). In van der Heide and de Witte (2016) and van der Heide et al. (2017), this bias was classified as low since proportions of and reasons for missing data were similar across both groups. All other studies presented complete data.
- Measurements of outcomes bias—bias of measurement of outcomes was considered low for (Rahman et al., 2007; Iwamuro et al., 2008; Bastiaens et al., 2011; Ambrosini et al., 2014; van der Heide et al., 2017; Estilow et al., 2018), given the use of devices for outcome measures assessment. The risk of bias was considered moderate in Sanchez et al. (2004); Jan Burgers (2015); Kooren et al. (2015); Peters et al. (2017), where the outcome measure assessment is computed by a therapists, who was considered to be only minimally influenced by knowledge of the intervention received by participants. Finally the risk of bias has been classified as serious in Lund et al. (2009); Gunn et al. (2016); van der Heide and de Witte (2016); Shank (2017), since these studies reported self-perceived outcome measures, with participants obviously aware of the intervention received.
- Selection of reported results bias—this risk of bias was considered low for Gunn et al. (2016); Shank (2017), which involved a number of patients higher than 20, moderate for Rahman et al. (2007); van der Heide and de Witte (2016); Peters et al. (2017), which included a number of participants between 10 and 19, and serious for all other studies, with a number of subjects lower than 10, meaning a high risk of selective reporting.

### 3.4. Treatment Effect

A meta-analysis was run to quantitatively merge results of 14 studies involving a total of 184 participants. The AD effect in supporting activities of daily living was quantified as the difference in primary outcome measure performance while non-wearing/wearing the specific AD. The forest plot for all included studies is represented in Table 6. The pooled estimated effect size with a random effects model (Equation 5) resulted to be 1.06, with a 95% CI (Equation 10) raging from 0.76 to 1.36. The *z*-test with the null hypothesis that there was no statistically significant difference between the treatment (i.e., users using AD) and the control group (i.e., users not using AD) resulted to be Z = 6.88, with associated *p* < 0.0001. The obtained overall effect size demonstrates that the ADs statistically significantly improve the performance in activities of daily living in people with neuromuscular diseases, with a large effect size (Cohen, 1988).

**Table 6:** Forest plots of comparison: Treatment group (with assistive device) vs. control group (without assistive device).

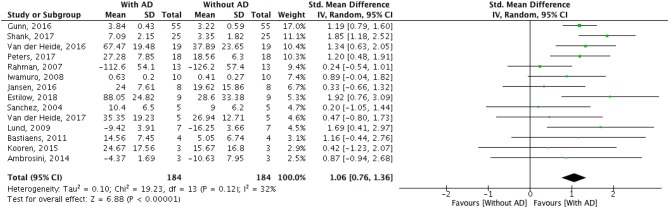

*Weights are computed with (Equation 6) and expressed as percentage*.

### 3.5. Acceptance

12/14 of the included studies reported no dropouts (Sanchez et al., 2004; Iwamuro et al., 2008; Lund et al., 2009; Bastiaens et al., 2011; Ambrosini et al., 2014; Jan Burgers, 2015; Kooren et al., 2015; Gunn et al., 2016; Peters et al., 2017; Shank, 2017; van der Heide et al., 2017; Estilow et al., 2018). The dropout rate was 23.5% in both the intervention and the control groups in Rahman et al. (2007), while it was equal to 5% and 13% in the control and treatment group respectively in van der Heide and de Witte (2016). Reported reasons for dropout included:

- Excessive contractures at the elbow and shoulder joints (Rahman et al., 2007);
- Interference with access to wheelchair joystick (Rahman et al., 2007);
- Absence of a caregiver who attached and removed the AD (Rahman et al., 2007);
- Sufficient functionality without the AD (Rahman et al., 2007);
- Difficulty in answering the required questions (van der Heide and de Witte, 2016).

None of the included studies reported adverse events during the intervention period, thus a pooled analysis was not performed.

### 3.6. Heterogeneity

*I*^2^ value associated with overall treatment effect was equal to 32%, meaning that the 32% of the total variability among effect sizes was caused by true heterogeneity between studies. The detected heterogeneity was investigated through sub-group analyses.

### 3.7. Sub-groups Analysis

#### 3.7.1. Comparison Between Studies Using Passive or Active ADs

The sub-groups analysis clustered the studies involving active devices and studies performed with passive devices, therefore investigating if the kind of device modifies the intervention effect (see Supplementary Material). The active devices group obtained an effect size 1.16 (95% *CI* = [0.73 1.60]) computed over 51 participants. The result of the *Z*-test of the null hypothesis that there was no effect was *Z*= 5.25 (*p* < 0.00001). The passive devices group included 133 subjects and it was characterized by an effect size of 0.99 (95% *CI* = [0.57 1.42]). The result of the *Z*-test was *Z*= 4.56 with an associated *p* < 0.00001. No differences between the two sub-groups have been detected (*I*^2^= 0%). This means that the type of device used (active or passive) does not modify the intervention effect.

#### 3.7.2. Comparison Between Studies With Self-Perceived and Externally-Assessed Outcome Measures

The sub-groups analysis clustered the studies involving the use of self-perceived outcome scores and studies performed using externally-assessed outcome measures, therefore investigating if the typology of outcome measure assessment modifies the intervention effect (see Figure S2). The sub-group including studies with self-perceived outcome measures was composed by 106 subjects and it was characterized by an effect size equals to 1.38 (95% *CI* = [1.08 1.68]). The result of the *Z*-test of the null hypothesis that there was no effect was *Z*= 8.92 (*p* < 0.00001). The sub-group including studies with externally-assessed outcome measures was composed by 78 subjects, and the effect size resulted to be 0.77 (95% *CI*= [0.41 1.11]). The result of the *Z*-test was *Z*= 4.40 with a *p* < 0.00001. The *I*^2^ index significantly decreases for both groups with respect to the general analysis: in the group of studies characterized by a self-perceived outcome measure, it was equal to 0%, while in the other group to 3%. These results indicate, respectively, absence and a very low level of heterogeneity. The test for sub-groups differences revealed a significant interaction, with *I*^2^ equals to 85.6% (95% *CI* = [42% 96.4%]). Therefore a statistically significant sub-groups effect was detected, and it can be derived that the considered covariate significantly modifies the treatment effect.

## 4. Discussion

### 4.1. Functional Improvement Mediated by the Use of ADs

Fourteen studies with 184 participants have been included in this meta-analysis, with the aim to assess the effectiveness of ADs in increasing activities of daily living and arm functions for people suffering from neuromuscular diseases (Table 7). All involved ADs improved patients ability to perform ADL in subjects affected by neuromuscular diseases. Different included studies were characterized by different changes, varying from an effect size of 0.24 (Rahman et al., 2007) to 1.92 (Estilow et al., 2018). The highest improvements were related to studies that used the range of motion as primary outcome measure (Estilow et al., 2018). However, this could implicate an extended working volume, even if not specifically related to daily life activities. In fact, studies characterized by functional outcome measures, such as the PUL (Kooren et al., 2015), which tested the impact of the AD when performing daily tasks, shown a lower enhancement of the performance indicator. Hedges *g* has been used to compute the standardized mean difference of upper limbs functionality with and without the support of AD for the different primary outcome measures identified. A random-effect model has been applied to obtain a weighted effect size across all included studies. We shown that ADs significantly increase the ability to perform daily life activities for people affected by neuromuscular diseases, with an associated effect size of 1.06 (*p* < 0.00001). According to Cohen (Cohen, 1988), this result represents a large effect size in favor of the use of ADs. Furthermore, adverse events and dropouts were uncommon and did not appear to be more frequent in participants when they used ADs. The use of ADs could be considered to be safe and acceptable to most participants included in the analyzed trials, which confirms the attitude of patients towards the use of the technologies (Mehrholz et al., 2015).

**Table 7:** Summary of findings table for the main comparisons.

**SUMMARY OF FINDINGS:**				
**Effectiveness of assistive devices for upper limb functionality for people with neuromuscular diseases**				
**Patient or population**: people affected by degenerative neuromuscular diseases				
**Setting**: rehabilitation facilities or patients' home				
**Intervention**: comparison between use and not use of assistive devices				
**Outcomes**	Anticipated absolute effects^*^ **(95% CI)**		**Number of subjects (studies)**	**Certainty of the evidence (GRADE)**
	**Assumed risk: ADL without assistive devices**	**Corresponding risk: ADL with assistive devices**		
**Activity of Daily Living**	ADL for the most representative study (Peters et al., 2017): SMD 0.28 (0.10)^*^	SMD **1.06** higher [0.76 to 1.36 higher]	184 (14 studies)	⊕⊕○○ LOW a,b,c
•**Activity of Daily Living:** subgroup analysis between active and passive devices. •**Active devices**	ADL for the most representative study (Peters et al., 2017): SMD 0.28 (0.10)^*^	SMD **1.16 higher** [0.71 to 1.62 higher]	94 (6 studies)	⊕⊕○○ LOW a,b,c
•**Activity of Daily Living**: subgroup analysis between active and passive devices. •**Passive devices**	ADL for the most representative study (Iwamuro et al., 2008): SMD 0.41 (0.27)^*^	SMD **1.01 higher** [0.61 to 1.41 higher]	274 (11 studies)	⊕⊕○○ LOW a,b,c
•**Activity of Daily Living**: subgroup analysis between self-perceived and externally assessed scales. •**Self-perceived scales**	ADL for the most representative study (van der Heide et al., 2017): SMD 0.38 (0.24)^*^	SMD **1.38 higher** [1.08 to 1.68 higher]	212 (4 studies)	⊕⊕⊕○ MODERATE a,b
•**Activity of Daily Living:** subgroup analysis between self-perceived and externally assessed scales. •**Externally assessed scales**	ADL for the most representative study (Peters et al., 2017): SMD 0.28 (0.10)^*^	SMD **0.77 higher** [0.42 to 1.11 higher]	156 (10 studies)	⊕⊕○○ LOW a,b,c

* *The study with the lowest risk of bias and the highest number of participants was chosen. Mean and SD are reported*.

*ADL, Activities of daily living; CI, Confidence interval; SD, Standard deviation; SMD, Standardized mean difference*.

GRADE working group grades of evidence:

*High: Further research is very unlikely to change our confidence in the estimate of effect*.

*Moderate: Further research is likely to have an important impact on our confidence in the estimate of effect and may change the estimate*.

*Low: Further research is very likely to have an important impact on our confidence in the estimate of effect and is likely to change the estimate*.

*Very low quality: We are very uncertain about the estimate*.

*Explanations:*.

*a. Risk of bias*.

*b. Imprecision of results*.

*c. Publication bias*.

*The four circles represent the maximum level of evidence (i.e., high, corresponding to four points). Each analysis is associated with its found level of evidence, represented by the circle marked with a cross. Indeed, “low” is represented by 2 points over 4, “moderate” 3 points over 4*.

### 4.2. Functional Improvement Mediated by the Use of Passive or Active ADs

From the studies included in the current systematic review, the use of active or passive devices seems not to influence the intervention effect i.e., they both improve the ability to perform activities of daily living in individuals with neuromuscular diseases. However, it has to be noted that different levels of disability have not been explicitly considered in the analysis. Users stratification with respect to current disability should be investigated to characterize the different users requirements, and consequent ADs technical requirements. In a previous pilot study, three categories have been identified, and namely mild, moderately, and severely impaired subjects, and the use of the same active and passive device has been investigated. Authors found that as disability level changes, the benefit from different devices changes as well. In particular, it has to be considered that severely impaired patients should use active devices that offer them a higher compensation and allow them to perform larger movements with less effort (Antonietti et al., 2019).

### 4.3. Functional Improvement Evaluated Through Externally-Assessed or Self-Perceived Outcome Measurements

When comparing studies including self-perceived scales or externally-assessed scales as outcome measures, the intervention effect presents a significant interaction. In particular, the effect size obtained when considering self-perceived scales has been shown to be almost double with respect to the effect size obtained from externally-assessed scales. This means that the patients' perception is often higher than the functional gain detectable through clinical scales or through a system measurement. Even if different from what has been objectively measured, it is important to take into consideration patients' evaluation, since it takes into account the effective effort required and the real benefit perceived by patients themselves. The two assessment methods give information with respect to a different, but equally important concept of the functional status of the patients. They are relevant in the same way and rigorous studies about the effects of a new AD should provide evaluation based on both kinds of scales.

### 4.4. Quality of Evidence

The quality of evidence ranged from low to moderate, as shown in Table 7. The analysis performed including all studies is characterized by a low level of evidence. This is due to the discussed risks of bias which downgraded the quality of evidence of three levels from high to very low, given some important limitations (i) in the selection of participants and in the blindness of outcome assessors, (ii) imprecision of results, due to the low number of studies and participants, and (iii) risk of publication bias (in 9 out of 14 studies). However, the large magnitude of effect and the presence of a clear dose-response gradient allowed the quality of evidence to be upgraded from very low to low. Same considerations are valid for the sub-groups involving active devices, passive devices and externally-assessed outcome measures, characterized by a low quality of evidence. Finally the sub-group involving self-perceived outcome measures shows a moderate level of evidence. In this case, in fact, only one study over four presented a serious risk of reporting bias, so this analysis did not show the publication bias. The level of evidence was reduced of two points due to risk of bias and imprecision of results and then upgraded of one level thanks to a large magnitude of effect and the presence of a clear dose-response gradient.

### 4.5. Overall Completeness and Applicability of Evidence

The results of this analysis seem to be quite generalizable, however, the following factors produce uncertainty:

- Only few studies that investigated the effect of ADs were found;
- Included studies show important limitations in terms of reduced number of participants and, especially, in the design of the trials themselves;
- The quality of evidence, except for one comparison, was rated as low.

In addition to the two possible sources of heterogeneity explored in this study, further aspects might influence the functional effect of AD in patients daily life. For example, different ADs might be differentially effective depending on the target pathology or on the specific level of disability of the user. Further methodologically proper studies which properly characterize included population are needed to explore these aspects.

### 4.6. Implications for Practice

People with neuromuscular diseases experience limitations while performing activities of daily living, in their independence and quality of life and they need to rely on assistance from caregivers. To compensate for the muscle weakness and for the impossibility of executing activity of daily living, they could benefit from the use AD. This meta-analysis demonstrated the efficacy of upper limb ADs for people suffering from neuromuscular diseases. The benefits have been demonstrated to be definitely higher than associated risks and therefore a strong recommendation, based on a level of evidence ranging from moderate to low, to use them is suggested.

### 4.7. Implications for Research

There is a need of well-designed, large-scale, multicenter research trials to enable appraisal and interpretation of results and to evaluate benefits and harms of AD in patients affected by neuromuscular diseases. Future studies should also investigate the most severely impaired people, who are not reflected so far in the existing trials. It is recommended to use both externally-assessed and self-perceived scales in clinical practice as well as for research purposes.

## 5. Conclusions

As far as we know, no other systematic reviews have been performed on the effects of ADs in improving daily life activities for patients with neuromuscular diseases. There is currently insufficient moderate/high quality of evidence to make conclusions about the benefits of ADs for improving activities of daily living in people affected by neuromuscular or neuromotor diseases. However, since it was not found evidence of side effects, further research into this type of therapy should be performed. It also has to be considered that ADs are expensive and they could create additional costs in patients' usual care. The general applicability of ADs might therefore be limited simply due to lack of access of these devices. All these points, taken together, might limit the applicability of this type of assistance in the day-to-day patients' routine.

## Data Availability Statement

All datasets generated for this study are included in the article/Supplementary Material.

## Author Contributions

MG and AP conceptualized the project. VL conducted the literature review, statistical analyses, and data syntheses. VL, AA, and MG conducted meta-analyses. All authors participated in the manuscript preparation with significant intellectual contributions.

### Conflict of Interest

The authors declare that the research was conducted in the absence of any commercial or financial relationships that could be construed as a potential conflict of interest.

## Footnotes

^1^https://www.cochranelibrary.com/central

^2^http://apps.webofknowledge.com/

^3^https://www.ncbi.nlm.nih.gov/pubmed/

^4^https://www.crd.york.ac.uk/prospero/

^5^https://www.pedro.org.au
