# Supplementary material for: The Effectiveness of Wearable Upper Limb Assistive Devices in Degenerative Neuromuscular Diseases: A Systematic Review and Meta-Analysis
Source: Front Bioeng Biotechnol. 2020 Jan 24;7:450. doi: 10.3389/fbioe.2019.00450 (PMC6992540; doi:10.3389/fbioe.2019.00450)
Supplement: Supplementary file 1 [file Data_Sheet_1.PDF]

Supplementary Material

1 SUPPLEMENTARY TABLES AND FIGURES

1.1 Figures

Sub-group analysis forest plots

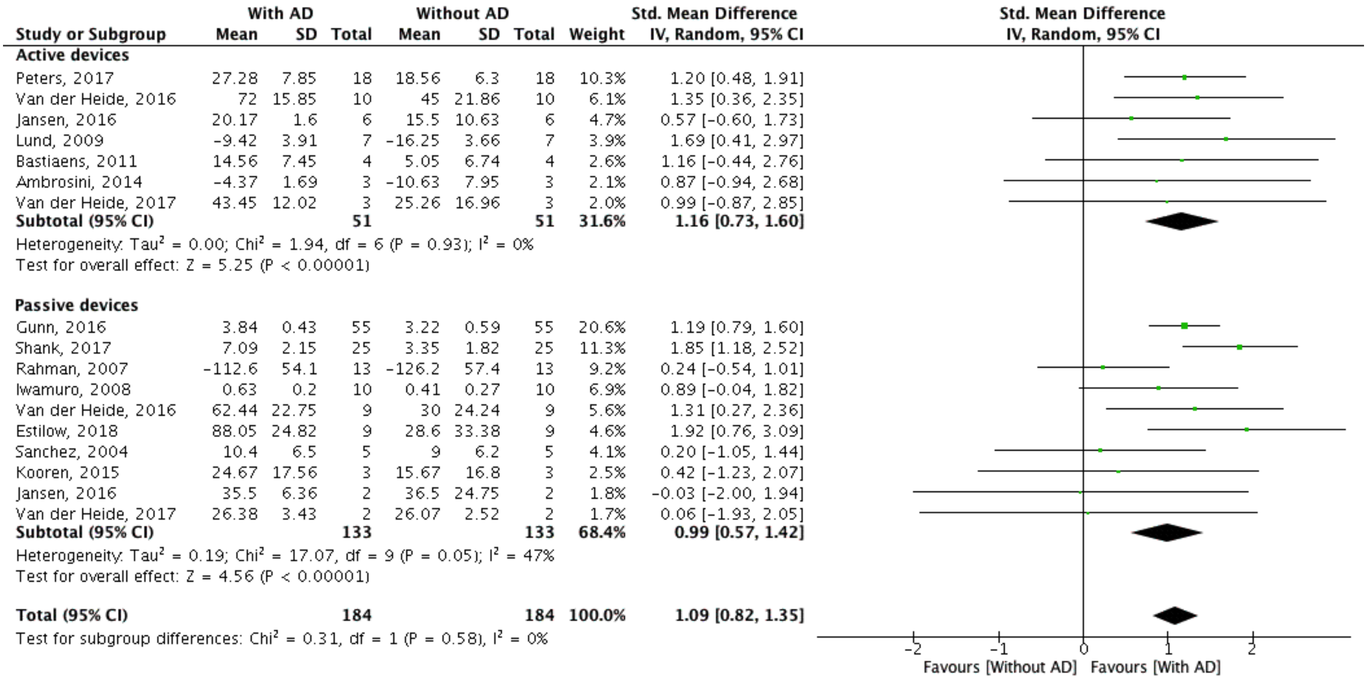

Study or Subgroup	With AD Mean	With AD SD	With AD Total	Without AD Mean	Without AD SD	Without AD Total	Weight	Std. Mean Difference IV, Random, 95% CI
**Active devices**								
Peters, 2017	27.28	7.85	18	18.56	6.3	18	10.3%	1.20 [0.48, 1.91]
Van der Heide, 2016	72	15.85	10	45	21.86	10	6.1%	1.35 [0.36, 2.35]
Jansen, 2016	20.17	1.6	6	15.5	10.63	6	4.7%	0.57 [−0.60, 1.73]
Lund, 2009	−9.42	3.91	7	−16.25	3.66	7	3.9%	1.69 [0.41, 2.97]
Bastiaens, 2011	14.56	7.45	4	5.05	6.74	4	2.6%	1.16 [−0.44, 2.76]
Ambrosini, 2014	−4.37	1.69	3	−10.63	7.95	3	2.1%	0.87 [−0.94, 2.68]
Van der Heide, 2017	43.45	12.02	3	25.26	16.96	3	2.0%	0.99 [−0.87, 2.85]
**Subtotal (95% CI)**			**51**			**51**	**31.6%**	**1.16 [0.73, 1.60]**
Heterogeneity: $Tau^2 = 0.00$; $Chi^2 = 1.94$, df = 6 (P = 0.93); $I^2 = 0\%$								
Test for overall effect: Z = 5.25 (P < 0.00001)								
**Passive devices**								
Gunn, 2016	3.84	0.43	55	3.22	0.59	55	20.6%	1.19 [0.79, 1.60]
Shank, 2017	7.09	2.15	25	3.35	1.82	25	11.3%	1.85 [1.18, 2.52]
Rahman, 2007	−112.6	54.1	13	−126.2	57.4	13	9.2%	0.24 [−0.54, 1.01]
Iwamuro, 2008	0.63	0.2	10	0.41	0.27	10	6.9%	0.89 [−0.04, 1.82]
Van der Heide, 2016	62.44	22.75	9	30	24.24	9	5.6%	1.31 [0.27, 2.36]
Estilow, 2018	88.05	24.82	9	28.6	33.38	9	4.6%	1.92 [0.76, 3.09]
Sanchez, 2004	10.4	6.5	5	9	6.2	5	4.1%	0.20 [−1.05, 1.44]
Kooren, 2015	24.67	17.56	3	15.67	16.8	3	2.5%	0.42 [−1.23, 2.07]
Jansen, 2016	35.5	6.36	2	36.5	24.75	2	1.8%	−0.03 [−2.00, 1.94]
Van der Heide, 2017	26.38	3.43	2	26.07	2.52	2	1.7%	0.06 [−1.93, 2.05]
**Subtotal (95% CI)**			**133**			**133**	**68.4%**	**0.99 [0.57, 1.42]**
Heterogeneity: $Tau^2 = 0.19$; $Chi^2 = 17.07$, df = 9 (P = 0.05); $I^2 = 47\%$								
Test for overall effect: Z = 4.56 (P < 0.00001)								
**Total (95% CI)**			**184**			**184**	**100.0%**	**1.09 [0.82, 1.35]**
Test for subgroup differences: $Chi^2 = 0.31$, df = 1 (P = 0.58), $I^2 = 0\%$								

Figure S1. Forest plots of comparison between active and passive devices: Treatment Group (with assistive device) vs. Control Group (without assistive device).

Study or Subgroup	With AD Mean	SD	Total	Without AD Mean	SD	Total	Weight	Std. Mean Difference IV, Random, 95% CI
**Self-perceived scales**								
Gunn, 2016	3.84	0.43	55	3.22	0.59	55	17.0%	1.19 [0.79, 1.60]
Shank, 2017	7.09	2.15	25	3.35	1.82	25	11.1%	1.85 [1.18, 2.52]
Van der Heide, 2016	67.47	19.48	19	37.89	23.65	19	10.4%	1.34 [0.63, 2.05]
Lund, 2009	-9.42	3.91	7	-16.25	3.66	7	4.5%	1.69 [0.41, 2.97]
**Subtotal (95% CI)**			**106**			**106**	**43.0%**	**1.38 [1.08, 1.68]**
Heterogeneity: $Tau^2 = 0.00$; $Chi^2 = 2.92$, df = 3 (P = 0.40); $I^2 = 0\%$								
Test for overall effect: Z = 8.92 (P < 0.00001)								
**Externally assessed scales**								
Peters, 2017	27.28	7.85	18	18.56	6.3	18	10.3%	1.20 [0.48, 1.91]
Rahman, 2007	-112.6	54.1	13	-126.2	57.4	13	9.4%	0.24 [-0.54, 1.01]
Iwamuro, 2008	0.63	0.2	10	0.41	0.27	10	7.4%	0.89 [-0.04, 1.82]
Jansen, 2016	24	7.61	8	19.62	15.86	8	6.7%	0.33 [-0.66, 1.32]
Estilow, 2018	88.05	24.82	9	28.6	33.38	9	5.3%	1.92 [0.76, 3.09]
Sanchez, 2004	10.4	6.5	5	9	6.2	5	4.7%	0.20 [-1.05, 1.44]
Van der Heide, 2017	35.35	19.23	5	26.94	12.71	5	4.6%	0.47 [-0.80, 1.73]
Bastiaens, 2011	14.56	7.45	4	5.05	6.74	4	3.1%	1.16 [-0.44, 2.76]
Kooren, 2015	24.67	17.56	3	15.67	16.8	3	2.9%	0.42 [-1.23, 2.07]
Ambrosini, 2014	-4.37	1.69	3	-10.63	7.95	3	2.5%	0.87 [-0.94, 2.68]
**Subtotal (95% CI)**			**78**			**78**	**57.0%**	**0.77 [0.42, 1.11]**
Heterogeneity: $Tau^2 = 0.01$; $Chi^2 = 9.24$, df = 9 (P = 0.42); $I^2 = 3\%$								
Test for overall effect: Z = 4.40 (P < 0.0001)								
**Total (95% CI)**			**184**			**184**	**100.0%**	**1.06 [0.76, 1.36]**
Test for subgroup differences: $Chi^2 = 6.94$, df = 1 (P = 0.008), $I^2 = 85.6\%$								

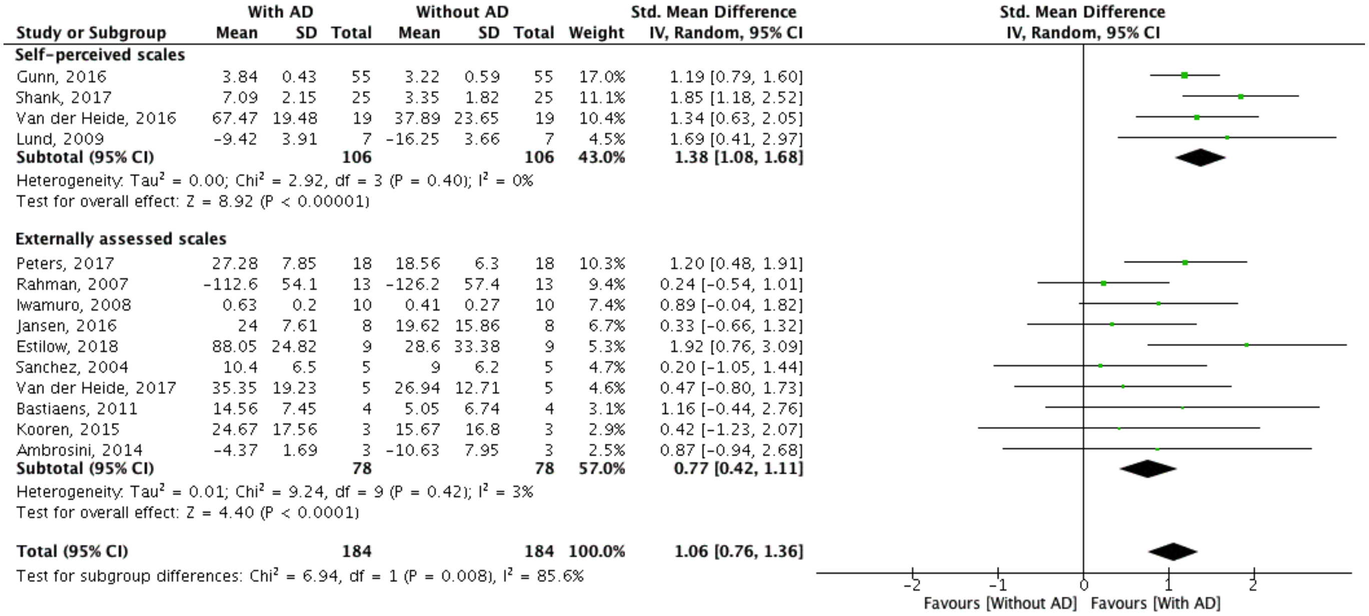

Figure S2. Forest plots of comparison between externally assessed and self-perceived scales: Treatment Group (with assistive device) vs. Control Group (without assistive device).

1.2 Tables

Details of clinical studies included in the meta-analysis

Table S1. Ambrosini et al. (2014)

**Methods**	Cohort study
**Participants**	Sample size: 10<br>Diseases: Spinal cord injuries
**Intervention**	8 repetitions of elbow flexion-extension with and without myocontrolled-NMES support, while tracking a trapezoidal target.<br>Active device: NMES and passive exoskeleton
**Outcome**	Root Mean Square Error (RMSE)

**Bias**	**Author's judgment**	**Support for judgment**
**Confounding**	Low risk	No confounding expected
**Selection of participants**	Moderate risk	Selection into the study may have been related to intervention and outcome
**Classification**	Low risk	Intervention status is well defined
**Deviations**	Low risk	There were no deviations from the intended interventions
**Missing data**	Low risk	Data were reasonably complete
**Measurement of outcomes**	Low risk	The outcomes were measured using a goniometer (Biometrics Ltd.) so the outcome measure is unlikely to be influenced by the knowledge of the intervention received by study participants
**Selection of result**	Serious risk	Number of subjects included in the study lower than 10

Table S2. Bastiaens et al. (2011)

**Methods**	Cohort study
**Participants**	Sample size: 4<br>Diseases: Multiple sclerosis
**Intervention**	Starting from a standardized middle position, move the arm as far as possible in 3 directions: forward, lateral and upward<br>Active device: HapticMaster and Sling
**Outcome**	Range Of Motion (ROM)

**Bias**	**Author's judgment**	**Support for judgment**
**Confounding**	Low risk	No confounding expected
**Selection of participants**	Moderate risk	Selection into the study may have been related to intervention and outcome
**Classification**	Low risk	Intervention status is well defined
**Deviations**	Low risk	There were no deviations from the intended interventions
**Missing data**	Low risk	Data were reasonably complete
**Measurement of outcomes**	Low risk	The outcomes were measured using an optic system so the outcome measure is unlikely to be influenced by the knowledge of the intervention received by study participants
**Selection of result**	Serious risk	Number of subjects included in the study lower than 10

Table S3. Estilow et al. (2018)

**Methods**	Cohort study
**Participants**	Sample size: 9<br>Diseases: Duchenne Muscular Dystrophy<br>Inclusion criteria: (1) confirmed diagnosis of DMD, (2) Brooke Upper Extremity Scale score between 2 and 5, and (3) wheelchair dependent
**Intervention**	Elevation of the arm at the shoulder and elbow joint and back to the starting position. Evaluation performed with and without arm support during the same day.<br>Passive device: Wrex
**Outcome**	Active Range Of Motion (AROM)

**Bias**	**Author's judgment**	**Support for judgment**
**Confounding**	Low risk	No confounding expected
**Selection of participants**	Low risk	All participants who would have been eligible for the target trial were included in the study
**Classification**	Low risk	Intervention status is well defined
**Deviations**	Low risk	There were no deviations from the intended interventions
**Missing data**	Low risk	Data were reasonably complete
**Measurement of outcomes**	Low risk	The outcomes were measured through goniometery so the outcome measure is unlikely to be influenced by the knowledge of the intervention received by study participants
**Selection of result**	Serious risk	Number of subjects included in the study lower than 10

Table S4. Gunn et al. (2016)

**Methods**	Cohort study
**Participants**	Sample size: 55<br>Diseases: Arthrogryposis Multiplex Congenital, Cerebral Palsy, Muscular Dystrophy, Spinal Muscular Atrophy, others<br>Inclusion criteria: (1) had used the device in the last 3 years, and (2) were less than 18 years of age when first fitted with the device
**Intervention**	During the same session respond to ten questions on personal functional ability with and without the AD after having used it for the last two to four years.<br>Passive device: Wrex
**Outcome**	Five-point Likert Scale

**Bias**	**Author's judgment**	**Support for judgment**
**Confounding**	Serious risk	Serious residual confounding expected
**Selection of participants**	Moderate risk	Selection into the study may have been related to intervention and outcome
**Classification**	Low risk	Intervention status is well defined
**Deviations**	Low risk	No information is reported on whether there is deviation from the intended intervention
**Missing data**	Low risk	Data were reasonably complete
**Measurement of outcomes**	Serious risk	The outcome measure was subjective
**Selection of result**	Low risk	Number of subjects included in the study higher than 20

Table S5. Iwamuro et al. (2008)

**Method**	Cohort study
**Participants**	Sample size: 10<br>Diseases: Chronic hemiparesis after stroke<br>Inclusion criteria: (1) at least 3 months post-stroke, (2) with 1 impaired upper extremity, characterized as stage 2 to stage 3 on the Chedoke-McMaster Stroke Assessment scale for the arm, and (3) not participating in a current rehabilitation therapy program
**Intervention**	36 reaching trials towards 12 targets positioned at the edge of the arm workspace of each subject with and without arm support during two sessions on separate days.<br>Passive device: T-Wrex
**Outcome**	Fraction of full reach toward the target (FOR)

**Bias**	**Author's judgment**	**Support for judgment**
**Confounding**	Low risk	No confounding expected
**Selection of participants**	Moderate risk	Selection into the study may have been related to intervention and outcome
**Classification**	Low risk	Intervention status is well defined
**Deviations**	Low risk	There were no deviations from the intended interventions
**Missing data**	Low risk	Data were reasonably complete
**Measurement of outcomes**	Moderate risk	The method of outcome assessment was comparable across intervention groups and the outcome measure was unlikely to be influenced by knowledge of the intervention received by study participants
**Selection of result**	Serious risk	Number of subjects included in the study lower than 10

Table S6. Jan Burgers (2015)

**Methods**	Cohort study
**Participants**	Sample size: 8
	Active device: Top/Help<br>Inclusion criteria: (1) wheelchair dependent, (2) confirmed diagnosis of DMD, (3) unable to touch the top of their head with at least one hand, and (4) able to use hands for tabletop activities
**Intervention**	19 items of grasp, grip, pinch, and gross movements evaluated with and without arm support during the same session.<br>Active (Top/Help Electrical) or Passive (Top/Help Mechanical)
**Outcome**	Action Research Arm Test (ARAT)

**Bias**	**Author's judgment**	**Support for judgment**
**Confounding**	Low risk	No confounding expected
**Selection of participants**	Moderate risk	Selection into the study may have been related to intervention and outcome
**Classification**	Low risk	Intervention status is well defined
**Deviations**	Low risk	There were no deviations from the intended interventions
**Missing data**	Low risk	Data were reasonably complete
**Measurement of outcomes**	Moderate risk	The method of outcome assessment was comparable across intervention groups and the outcome measure was unlikely to be influenced by knowledge of the intervention received by study participants
**Selection of result**	Serious risk	Number of subjects included in the study lower than 10

Table S7. Kooren et al. (2015)

**Methods**	Cohort study
**Participants**	Sample size: 3<br>Diseases: Duchenne Muscular Dystrophy
**Intervention**	Standardized single joint movements of shoulder and elbow and ADL tasks extracted from the “Performance of the Upper Limb Scale”<br>Passive device: A-Gear
**Outcome**	Performance of the Upper Limb (PUL)

**Bias**	**Author’s judgment**	**Support for judgment**
**Confounding**	Low risk	No confounding expected
**Selection of participants**	Moderate risk	Selection into the study may have been related to intervention and outcome
**Classification**	Low risk	Intervention status is well defined
**Deviations**	Low risk	There were no deviations from the intended interventions
**Missing data**	Low risk	Data were reasonably complete
**Measurement of outcomes**	Moderate risk	The method of outcome assessment was comparable across intervention groups and the outcome measure was unlikely to be influenced by knowledge of the intervention received by study participants
**Selection of result**	Serious risk	Number of subjects included in the study equal to 10

Table S8. Lund et al. (2009)

**Methods**	Cohort study
**Participants**	Sample size: 7<br>Diseases: Amyotrophic Lateral Sclerosis, Arthrogryposis Multiplex Congenita, Post Traumatic Dystrophy, Spinal Muscular Atrophy<br>Inclusion criteria: (1) no cognitive impairments
**Intervention**	During the same interview rate the difficulty of performing 7 activities considered important by the patient himself with and without arm support<br>Active device: ARMON
**Outcome**	Individually Prioritized Problem Assessment (IPPA)

**Bias**	**Author's judgment**	**Support for judgment**
**Confounding**	Serious risk	Serious residual confounding expected
**Selection of participants**	Moderate risk	Selection into the study may have been related to intervention and outcome
**Classification**	Low risk	Intervention status is well defined
**Deviations**	Low risk	There were no deviations from the intended interventions
**Missing data**	Low risk	Data were reasonably complete
**Measurement of outcomes**	Serious risk	The outcome measure was subjective
**Selection of result**	Serious risk	Number of subjects included in the study lower than 10

Table S9. Peters et al. (2017)

**Methods**	Cohort study
**Participants**	Sample size: 18<br>Diseases: stroke<br>Inclusion criteria: (1) volitionally activated paretic biceps brachii EMG amplitude >= 5uV, (2) 1 stroke experience more than 12 months prior to study enrollment, (3) Mini-Mental State Examination score >= 24, (4) age between 21 and 80, (5) medically stable, (6) active shoulder abduction >= 20and active shoulder flexion =>30, and (7) more than a trace of Manual Muscle Testing (1/5) in biceps/triceps
**Intervention**	4 functional tasks turning on a light switch, lifting a laundry basket bilaterally, bringing a spoon to the mouth and drinking from a cup evaluated with and without arm support during two sessions on the same day.<br>Active device: EMG signal (MyoPro Motion-G) that controls a powered orthosis
**Outcome**	Upper Extremity Section of the Fugl-Meyer Scale

**Bias**	**Author's judgment**	**Support for judgment**
**Confounding**	Low risk	No confounding expected
**Selection of participants**	Low risk	All participants who would have been eligible for the target trial were included in the study
**Classification**	Low risk	Intervention status is well defined
**Deviations**	Low risk	There were no deviations from the intended interventions
**Missing data**	Low risk	Data were reasonably complete
**Measurement of outcomes**	Moderate risk	The method of outcome assessment was comparable across intervention groups and the outcome measure was unlikely to be influenced by knowledge of the intervention received by study participants
**Selection of result**	Moderate risk	Number of subjects included in the study between 10 and 20

Table S10. Rahman et al. (2007)

**Methods**	Cohort study
**Participants**	Sample size: 13<br>Disease: Becker Muscular Dystrophy, Congenital Muscular Dystrophy, Duchenne Muscular Dystrophy, Spinal Muscular Atrophy<br>Inclusion criteria: (1) arm strength of less than 5 on the Manual Muscle Test scale, and (2) wheelchair dependent
**Intervention**	7 activities of daily living writing, turning cards, manipulating small objects, feeding, stacking checkers, lifting large light objects and lifting large heavy objects. Evaluation was performed first without the arm support and then with it after 2 weeks of home use.<br>Passive device: Wrex
**Outcome**	Jebsen Taylor Hand Function Timed Test

**Bias**	**Author's judgment**	**Support for judgment**
**Confounding**	Low risk	No confounding expected
**Selection of participants**	Moderate risk	Selection into the study may have been related to intervention and outcome
**Classification**	Low risk	Intervention status is well defined
**Deviations**	Moderate risk	There were deviations from intended intervention since few patients did not practice at home with the AD, but their impact on the outcome is expected to be slight
**Missing data**	Moderate risk	Proportion of and reason for missing participants differ slightly across intervention groups
**Measurement of outcomes**	Low risk	The method of outcome assessment was comparable across intervention groups and the outcome measure was unlikely to be influenced by knowledge of the intervention received by study participants
**Selection of result**	Moderate risk	Number of subjects included in the study between 10 and 20

Table S11. Sanchez et al. (2004)

**Methods**	Cohort study
**Participants**	Sample size: 5<br>Disease: chronic stroke
**Intervention**	14 functional tasks evaluated with and without arm support during the same session.<br>Passive device: T-Wrex
**Outcome**	Upper Extremity Section of the Fugl-Meyer Scale

**Bias**	**Author's judgment**	**Support for judgment**
**Confounding**	Low risk	No confounding expected
**Selection of participants**	Moderate risk	Selection into the study may have been related to intervention and outcome
**Classification**	Low risk	Intervention status is well defined
**Deviations**	Low risk	There were no deviations from the intended interventions
**Missing data**	Low risk	Data were reasonably complete
**Measurement of outcomes**	Moderate risk	The method of outcome assessment was comparable across intervention groups and the outcome measure was unlikely to be influenced by knowledge of the intervention received by study participants
**Selection of result**	Serious risk	Number of subjects included in the study lower than 10

Table S12. Shank (2017)

**Methods**	Cohort study
**Participants**	Sample size: 25<br>Diseases: Arthrogryposis Multiplex Congenital, Cerebral Palsy, Muscular Dystrophy, Spinal Muscular Atrophy, others<br>Inclusion criteria: (1) arm weakness between 1 and 3 on the Manual Muscle Test, (2) passive elbow range of motion >= 50, and (3) passive shoulder flexion >= 90
**Intervention**	During the same interview rate the ability to perform identified important tasks with and without arm support.<br>Passive device: Wrex
**Outcome**	Canadian Occupational Performance Measure (COPM)

**Bias**	**Author's judgment**	**Support for judgment**
**Confounding**	Serious risk	Serious residual confounding expected
**Selection of participants**	Moderate risk	Selection into the study may have been related to intervention and outcome
**Classification**	Low risk	Intervention status is well defined
**Deviations**	Low risk	No information is reported on whether there is deviation from the intended intervention
**Missing data**	Low risk	Data were reasonably complete
**Measurement of outcomes**	Serious risk	The outcome measure was subjective
**Selection of result**	Low risk	Number of subjects included in the study higher than 20

Table S13. van der Heide and de Witte (2016)

**Methods**	Cross sectional study
**Participants**	Sample size: 19<br>Diseases: Amyotrophic Lateral Sclerosis, Ehlers-Danlos Syndrome, Limb-Girdle Muscular Dystrophy, Multifocal Motor Neuropathy, Multiple Sclerosis, Spinal Cord Injury, Spinal Muscular Atrophy
**Intervention**	During the same interview rate the ability to perform activities of daily living with and without arm support.<br>Active or passive devices: Armon Edero (A), Armon Ayura (A), Darwing (A), Balancer (P)
**Outcome**	Perceived Functional Benefit (VAS scale)

**Bias**	**Author's judgment**	**Support for judgment**
**Confounding**	Low risk	No confounding expected
**Selection of participants**	Moderate risk	Selection into the study may have been related to intervention and outcome
**Classification**	Low risk	Intervention status is well defined
**Deviations**	Low risk	There were no deviations from the intended interventions
**Missing data**	Low risk	Proportions of and reasons for missing participants were similar across intervention groups
**Measurement of outcomes**	Serious risk	The outcome measure was subjective
**Selection of result**	Moderate risk	Number of subjects included in the study between 10 and 20

Table S14. van der Heide et al. (2017)

**Methods**	Cohort study
**Participants**	Sample size: 5<br>Diseases: Amyotrophic Lateral Sclerosis, Muscular Dystrophy, Stroke, Spinal Stenosis<br>Inclusion criteria: (1) use of the arm support in the previous week, (2) age >18, and (3) no cognitive impairments
**Intervention**	9 functional tasks touching the ipsilateral ear, eating with a spoon, drinking from a glass, touching the opposite axilla, touching the seat between the upper legs, combing hair, stroking a pet, grabbing a door handle and Grabbing a book from the shelf. Evaluation performed with and subsequentially without arm support.<br>Active or passive devices: Top/Help (A), Sling (P)
**Outcome**	Range Of Motion (ROM)

**Bias**	**Author's judgment**	**Support for judgment**
**Confounding**	Low risk	No confounding expected
**Selection of participants**	Serious risk	Quote: "Participants were selected on the basis of convenience sampling"
**Classification**	Low risk	Intervention status is well defined
**Deviations**	Low risk	There were no deviations from the intended interventions
**Missing data**	Low risk	Proportions of and reasons for missing participants were similar across intervention groups
**Measurement of outcomes**	Low risk	The outcomes were measured using the MMAAS motion capturing instrument so the outcome measure is unlikely to be influenced by the knowledge of the intervention received by study participants
**Selection of result**	Serious risk of bias	Number of subjects included in the study lower than 10

REFERENCES

Ambrosini E, Ferrante S, Schauer T, Klauer C, Gaffuri M, Ferrigno G, et al. A myocontrolled neuroprosthesis integrated with a passive exoskeleton to support upper limb activities. *Journal of Electromyography and Kinesiology: Official Journal of the International Society of Electrophysiological Kinesiology* **24** (2014) 307–317. doi:10.1016/j.jelekin.2014.01.006.

Bastiaens H, Alders G, Feys P, Notelaers S, Coninx K, Kerkhofs L, et al. Facilitating robot-assisted training in MS patients with arm paresis: a procedure to individually determine gravity compensation. *IEEE ... International Conference on Rehabilitation Robotics: [proceedings]* **2011** (2011) 5975507. doi:10.1109/ICORR.2011.5975507.

Estilow T, Glanzman AM, Powers K, Moll A, Flickinger J, Medne L, et al. Use of the Wilmington Robotic Exoskeleton to Improve Upper Extremity Function in Patients With Duchenne Muscular Dystrophy. *The American Journal of Occupational Therapy: Official Publication of the American Occupational Therapy Association* **72** (2018) 7202345010p1–7202345010p5. doi:10.5014/ajot.2018.022939.

Gunn M, Shank TM, Eppes M, Hossain J, Rahman T. User Evaluation of a Dynamic Arm Orthosis for People With Neuromuscular Disorders. *IEEE transactions on neural systems and rehabilitation engineering: a publication of the IEEE Engineering in Medicine and Biology Society* **24** (2016) 1277–1283. doi:10.1109/TNSRE.2015.2492860.

Iwamuro BT, Cruz EG, Connelly LL, Fischer HC, Kamper DG. Effect of a gravity-compensating orthosis on reaching after stroke: evaluation of the Therapy Assistant WREX. *Archives of Physical Medicine and Rehabilitation* **89** (2008) 2121–2128. doi:10.1016/j.apmr.2008.04.022.

Jan Burgers MJ. Upper Limb Training with Dynamic Arm Support in Boys with Duchenne Muscular Dystrophy: A Feasibility Study. *International Journal of Physical Medicine and Rehabilitation* **03** (2015). doi:10.4172/2329-9096.1000256.

Kooren PN, Dunning AG, Janssen MMHP, Lobo-Prat J, Koopman BFJM, Paalman MI, et al. Design and pilot validation of A-gear: a novel wearable dynamic arm support. *Journal of NeuroEngineering and Rehabilitation* **12** (2015) 83. doi:10.1186/s12984-015-0072-y.

Lund K, Brandt R, Gelderblom GJ, Herder JL. A user-centered evaluation study of a mobile arm support. *2009 IEEE International Conference on Rehabilitation Robotics* (Kyoto, Japan: IEEE) (2009), 582–587. doi:10.1109/ICORR.2009.5209494.

Peters HT, Page SJ, Persch A. Giving Them a Hand: Wearing a Myoelectric Elbow-Wrist-Hand Orthosis Reduces Upper Extremity Impairment in Chronic Stroke. *Archives of Physical Medicine and Rehabilitation* **98** (2017) 1821–1827. doi:10.1016/j.apmr.2016.12.016.

Rahman T, Sample W, Seliktar R, Scavina MT, Clark AL, Moran K, et al. Design and testing of a functional arm orthosis in patients with neuromuscular diseases. *IEEE transactions on neural systems and rehabilitation engineering: a publication of the IEEE Engineering in Medicine and Biology Society* **15** (2007) 244–251. doi:10.1109/TNSRE.2007.897026.

Sanchez R, Reinkensmeyer D, Shah P, Liu J, Rao S, Smith R, et al. Monitoring functional arm movement for home-based therapy after stroke. *Conference proceedings: ... Annual International Conference of the IEEE Engineering in Medicine and Biology Society. IEEE Engineering in Medicine and Biology Society. Annual Conference* **7** (2004) 4787–4790. doi:10.1109/IEMBS.2004.1404325.

Shank. Outcome Measures with COPM of Children using a Wilmington Robotic Exoskeleton. *The Open Journal of Occupational Therapy* **5** (2017).

van der Heide L, de Witte L. The perceived functional benefit of dynamic arm supports in daily life. *Journal of Rehabilitation Research and Development* **53** (2016) 1139–1150. doi:10.1682/JRRD.2015.06.0099.

van der Heide LA, Ramakers I, Essers JM, Gelderblom GJ, de Witte LP, Meijer K. Is it possible to assess the effects of dynamic arm supports on upper extremity range of motion during activities of daily living in the domestic setting using a portable motion capturing device? – A pilot study. *Technology and Disability* **29** (2017) 91–99. doi:10.3233/TAD-160157.
